# Understanding Ammonium Transport in Bioelectrochemical Systems towards its Recovery

**DOI:** 10.1038/srep22547

**Published:** 2016-03-03

**Authors:** Ying Liu, Mohan Qin, Shuai Luo, Zhen He, Rui Qiao

**Affiliations:** 1 Department of Mechanical Engineering, Virginia Polytechnic Institute and State University, Blacksburg, VA 24061, USA; 2 Department of Civil and Environmental Engineering, Virginia Polytechnic Institute and State University, Blacksburg, VA 24061, USA

## Abstract

We report an integrated experimental and simulation study of ammonia recovery using microbial electrolysis cells (MECs). The transport of various species during the batch-mode operation of an MEC was examined experimentally and the results were used to validate the mathematical model for such an operation. It was found that, while the generated electrical current through the system tends to acidify (or basify) the anolyte (or catholyte), their effects are buffered by a cascade of chemical groups such as the NH_3_/NH_4_^+^ group, leading to relatively stable pH values in both anolyte and catholyte. The transport of NH_4_^+^ ions accounts for ~90% of the total current, thus quantitatively confirming that the NH_4_^+^ ions serve as effective proton shuttles during MEC operations. Analysis further indicated that, because of the Donnan equilibrium at cation exchange membrane-anolyte/catholyte interfaces, the Na^+^ ion in the anolyte actually facilitates the transport of NH_4_^+^ ions during the early stage of a batch cycle and they compete with the NH_4_^+^ ions weakly at later time. These insights, along with a new and simple method for predicting the strength of ammonia diffusion from the catholyte toward the anolyte, will help effective design and operation of bioeletrochemical system-based ammonia recovery systems.

Nitrogen removal from wastewater is of great importance to protect receiving water from eutrophication^1^. The primary form of nitrogen in wastewater is ammonia (or as a product of decomposing organic nitrogen)^2^, and thus removing ammonia from municipal and industrial wastewater prior to discharge is obligatory. Ammonia can be removed from wastewater using biological or physicochemical methods such as nitrification and denitrification, ion exchange, air stripping, and anaerobic ammonium oxidation (anammox)^3^,^4^ Because ammonia nitrogen is a key fertilizer component for agricultural applications, recovery instead of removal of ammonia from wastewater can greatly impact on both sustainable wastewater management and economics. An emerging approach for recovering ammonia is through bioelectrochemical systems (BES)^5^,^6^.

In BES-based ammonia recovery systems, organic compounds in wastewater are oxidized by the exoelectrogens growing on an anode, producing a current through its external circuit^7^. When cation exchange membranes (CEMs) are used^8^,^9^,^10^,^11^ the current is carried by cations such as NH_4_^+^ ions moving from the anolyte into the catholyte through the CEM. The NH_4_^+^ ions, once transported into the catholyte, are converted into NH_3_ molecules because the catholyte is usually rendered basic by the production of OH^−^ ions therein^12^,^13^,^14^. Some of these NH_3_ molecules can then be recovered through gas aeration. This type of process has been demonstrated in various BES including microbial fuel cells (MFCs) and microbial electrolysis cells (MECs). In MECs, a higher current density would greatly enhance ammonia recovery^9^, and thus MECs with external power exhibit a better performance for ammonium recovery than MFCs^15^. It was reported that ammonia can be recovered from ammonium-rich wastewater, such as synthetic wastewater, urine, and swine wastewater^12^,^13^,^16^,^17^.

While a general picture of ammonia recovery using BES is emerging, some issues remain open. In particular, the transport of NH_4_^+^ ions and NH_3_ is not fully understood despite their essential role in ammonia recovery. Some studies indicate that NH_4_^+^ transport dominates the current across the CEM (i.e., for each electron passing through the external circuit, ~1 NH_4_^+^ ion moves from the anolyte into the catholyte through the CEM)^18^, while other studies suggest that NH_4_^+^ ions may carry only 40% of the total current^5^,^12^,^16^,^19^. In addition, whether NH_4_^+^ ions move through the CEM via diffusion or migration is not clear. A recent study suggests that the migration of NH_4_^+^ ions dominates over their diffusion through a comparison of the net NH_4_^+^ transport under the close and open circuit conditions^9^. However, this conclusion was based on the assumption that the diffusion of NH_4_^+^ ions under these conditions is the same, whose validity/generality is not yet clear. In a seminal simulation study in which key transport phenomena and chemical reaction within the BES are modeled comprehensively, it was found that the diffusion of NH_3_ from the catholyte to the anolyte can greatly reduce the efficiency of recovery^20^. However, the condition under which such diffusion is important has not been delineated yet. Finally, since many cations such as Na^+^ ions exist in the anolyte, for a given current through the BES, they may compete with NH_4_^+^ ions for transport across the CEM^11^,^16^,^21^. How strong such competition is and how it affects the ammonia recovery is not well understood.

Another open issue is how the pH in the anolyte and catholyte is regulated. Since the pH values in the anolyte/catholyte affect how the total nitrogen content is partitioned between its two forms (NH_4_^+^ ions and NH_3_), and the latter in turn affects the transport of NH_4_^+^ ions through the CEM and the recovery of NH_3_ through aeration, it is essential to regulate these pH effectively. Furthermore, regulating the anolyte pH is also essential for ensuring microbial activity and hence the reliable operation of BES^8^,^22^,^23^. Maintaining a basic environment with pH >9 in the catholyte facilitates the conversion of NH_4_^+^ ions into NH_3_, which is key for the effective recovery of ammonia by aeration. The effective control of the pH in the anolyte and the catholyte requires a thorough understanding of what governs the pH in these electrolytes^11^,^21^,^24^. While it is known that the generation of proton in the anolyte and hydroxide ions in the catholyte plays an important role in controlling the pH^11^,^12^,^20^,^21^,^24^,^25^, how and to what extent the transport of all ions and their chemical reactions with each other affect the pH is little understood.

Resolving the above issues using experimental study alone is difficult due to the challenge in assessing details of all transport processes in the BES. Numerical modeling can address this challenge, but most prior modeling of BES focused on power production and organic removal^26^,^27^,^28^,^29^,^30^. In the only comprehensive model for BES-based ammonia recovery, the transport and chemical reactions of major species in BES was studied at a steady state^20^. The predictions of this model agree qualitatively with experimental observations and they led to critical insight into the effects of current density and membrane properties on the ammonia recovery. However, the model has not been validated by detailed comparison with experiments, and studies based on this model did not cover the issues described above, e.g., the competition of inert ions (i.e., ions that do not react with other species within the system, e.g., the Na^+^ ions) with the NH_4_^+^ ions for transport across the CEM cannot be studied using steady state models. Here, we have studied the ammonia recovery in a representative BES – microbial electrolysis cells, by integrating experiments with simulations.

## Results

**Figure 1:**
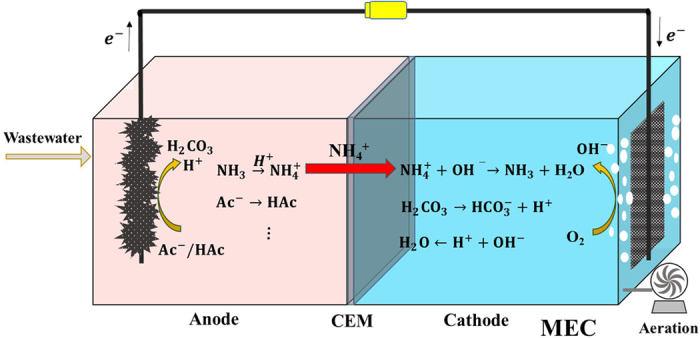
A sketch of the MEC-based ammonia recovery system.

A bench-scale cubic shape MEC was used for experimental data collection (see Fig. 1). The wastewater fed into the anode was synthetic digestion effluent of livestock waste while deionized water was used as catholyte. The anolyte was partially replaced (150 mL) every 48 h while the catholyte was unchanged during 3 batch cycles. The variation of organic matters, NH_4_^+^/NH_3_ concentration, pH and inert ions in both chambers were recorded for analysis and model validation.

To simulate the batch-mode operation of MECs for ammonia recovery, we extend the steady-state models developed earlier^20^. The models consider the mass conservation for all species (without loss of generality, the following species are included: Na^+^, Cl^−^, HAc, Ac^−^, NH_4_^+^, NH_3_, H_2_CO_3_, HCO_3_^−^, CO_3_^2−^, H^+^ and OH^−^), the transport of each species (diffusion and migration) across the membrane, the chemical reactions among different species, and gas-solution equilibrium. For chemical reactions, we consider mostly acid-base reactions which are essential for ammonia recovery (e.g., 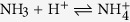, H_2_CO_3_ 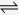 H^+^ + HCO_3_^−^, … and a full list of these reactions is provided in the Supplementary Information). Using the operation parameters (e.g., the anolyte/catholyte volume V_1_/V_2_, the membrane surface area *A,* etc.) and the measured current density as input, the model can predict the time evolution of the concentration of each species in the system (hereafter, the concentration of species *i* in anolyte/catholyte chamber is denoted as 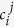, with *j* = 1 and 2 for anode and cathode chamber, respectively) and the recovery of ammonia by aeration. Below we first validate the mathematical model, then investigate the above issues and how they impact the ammonia recovery in the MEC.

### Model validation

**Figure 2:**
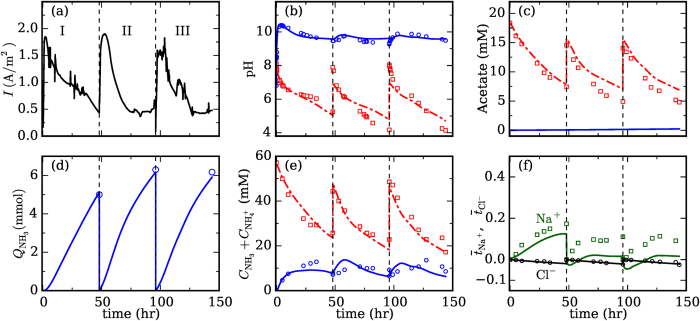
Comparison of experimental and model studies of ammonia recovery during three batch cycles of MEC operation. The current density measured experimentally (**a**) is feed into the models to predict the various observables of the anolyte and the catholyte: the pH (**b**), the acetate concentration (**c**), the amount of NH_3_ collected from catholyte by aeration (**d**), the total nitrogen content (**e**), and the transport of Na^+^ and Cl^−^ ions out of the anolyte (**f**). Symbols are the experiment data and lines are model prediction. In (**b–e**), red lines are for anolyte and blue lines are for catholyte. In (**c**), the acetate concentration in catholyte is below the detection limit of our equipment and thus not shown. In (**f**), to highlight the relative importance of Na^+^/Cl^−^ ion transport in the overall charge transport in the system, their transport is quantified using the cumulative transport number 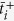 (see text, 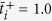 means that the charge carried by the transport a species *i* is equal to the total charge passed through the membrane). A detailed summary of operating conditions and parameters (e.g., initial species concentration in anolyte/catholyte) is in provided in Tables S2–S4 in SI.

We first validate the mathematical model for ammonia recovery using MEC by comparing its predictions against experimental data obtained under the same operation conditions. Ammonium recovery was firstly studied experimentally. During one batch cycle, the COD concentration decreased from 1089 ± 169 to 262 ± 83 mg L^−1^, resulting in a Coulombic efficiency (CE) of 34.7 ± 5.9%. The maximum current density was 1.89 A m^−2^ (Fig. 2a). In the anolyte, the pH decreased from 7.98 ± 0.08 to 4.52 ± 0.63; in the catholyte, the pH increased dramatically initially and then stabilized at ~9.7 (Fig. 2b). The acetate concentration in the anolyte decreased from 15.6 ± 2.4 to 5.4 ± 0.9 mM, and that in the catholyte was always below the detection limit of our equipment (Fig. 2c). The latter is consistent with the fact that the diffusion loss of acetate through CMI-7000 is small^21^. Experimentally, the total input nitrogen as ammonium in each batch cycle was 9.3 ± 0.4 mmol, among which 35.7 ± 7.4% stayed in the anolyte, 0.9 ± 0.3% remained in the catholyte and 66.2 ± 2.7% was stripped out as ammonia and absorbed by the sulfuric acid. In each batch cycle (2 days), 6.1 ± 0.1 mmol of NH_3_ gas was collected through aeration, resulting in a recovery rate of 10.2 ± 0.1 g_N_ m^−2^ d^−1^ (vs. membrane surface area)^18^. Ammonia oxidation in the anode chamber might not occur because we did not detect any nitrite or nitrate in the anolyte. Numerically, the simulation predicted the total ammonia concentration in anolyte and catholyte to be 5% and 18.4% within the corresponding maximum measured values, respectively. Some Na^+^ and Cl^−^ ions crossed into the catholyte through the CEM in each cycle. To assess their role in the net charge transport through the CEM, we computed the cumulative transport number 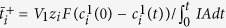 for these ions during each cycle. At the end of each cycle, 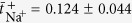 and 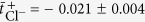 (Fig. 2f), indicating that overall the transport of these ions contributes to ~10% of the total current. Since the transport of all other ions except 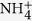 ions through the CEM is very small, we thus conclude that the transport of 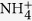 ions through the CEM accounts for ~90% of the total current in our system, which is consistent with some earlier studies^18^.

The above ammonia recovery operation was also simulated using the mathematical model developed. As shown in Fig. 2b–f, the model predictions agree quite well with the experimental data. In particular, the model captures the evolution of pH, acetate concentration, and total nitrogen content in the anolyte/catholyte. The recovery of NH_3_ gas was also accurately captured. The model underestimates the transport of Na^+^ ions across the CEM (Fig. 2f). However, given that the transport of Na^+^ ions accounts for only a small fraction of the total current through the CEM and these ions do not interact with other species in the system, these small deviations are deemed acceptable.

Using the validated model, we next examine the mechanisms of MEC-based ammonia recovery using simulations and address three issues outlined earlier, i.e., the pH regulation, NH_4_^+^ transport and ion competition in MECs. The initial conditions in the anolyte are the same as those in the above experiment. The catholyte initially features 10 mM of Na^+^ ions and accompanying carbonate group ions, which together produces a pH of ~6.8, as commonly found in experiments. Without a loss of generality, the system operates at a fixed current density of I = 1 A m^−2^ for 48 hours.

### pH Regulation

To understand how the pH is regulated, we note that, by considering the mass conservation of proton, its concentration evolution in the anolyte follows (for details, see Supplementary Information):


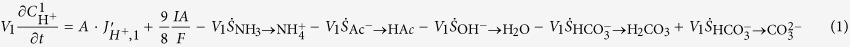


This equation shows that the proton concentration in the anolyte is affected by several factors: the transport of proton into or out of the anolyte through the CEM (the first term on the right side), the production of proton through Faraday reactions (the second term on right), and the consumption or production of protons by various chemical reactions (the remaining terms on right), e.g., 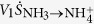 stands for the consumption of proton by the reaction 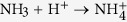. For brevity, the last two terms in Eq. (1) are hereafter lumped as 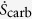.

**Figure 3:**
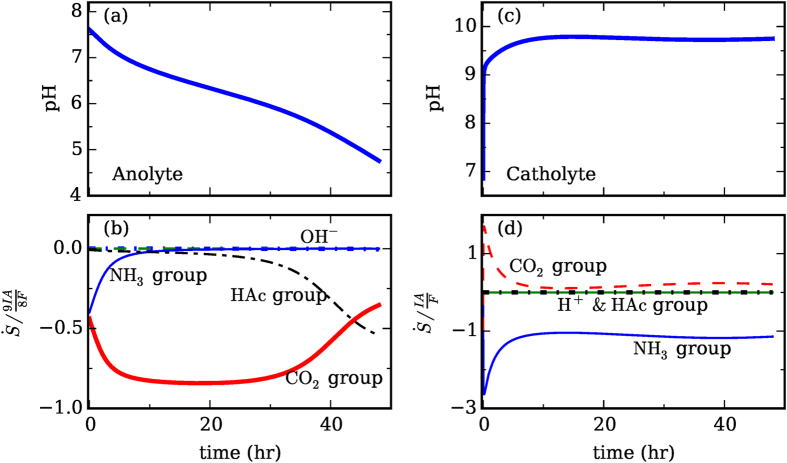
Regulation of pH in MEC during ammonia recovery operation. The variation of the pH values in the anolyte (**a**) and the catholyte (**c**) is governed by various Faradaic and chemical reactions (**b**,**d**) identified in Eq. (1,2). The generation/consumption of proton and hydroxide by the chemical reactions involving various ion groups in the anolyte and catholyte are shown in (**b**,**d**), respectively. These generation/consumption terms are normalized by the production of proton (in anolyte, for panel (b)) and hydroxide (in catholyte, for panel (d)) generated through Faradic reactions. [Operating conditions and parameters in this study are the same as in Fig. 2 except the followings: the current is I = 1 A/m^2^; the catholyte initially contains 10 mM of Na^+^ ions and the accompanying carbonate group ions to produce a pH of ~6.8].

To understand how the pH in the anolyte is regulated, we simulated the operation of MEC under a constant current of I = 1 A m^−2^. Figure 3a shows that the pH in the anolyte decreases steadily during the operation. To assess the relative importance of the various factors identified above in regulating the pH, we examined each term on the right-hand side of Eq. (1) and the results are shown in Fig. 3b (all terms are normalized by the Faradaic reaction term). We observe that most of the protons generated by the Faradaic reaction were neutralized by their reactions with NH_3_, the 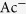 ions, and the carbonate ions. Hence, these chemical groups essentially serve as the “buffer” to keep the pH from changing dramatically. The relative contribution of these chemical groups in regulating the pH evolves during the operation of MEC: NH_3_ lost its buffer capability soon after the pH is below 7, while the carbonate ions remain effective in buffering the anolyte till ~40 hrs.

A similar equation for the evolution of the hydroxide concentration in the catholyte was derived to understand the regulation of pH therein:


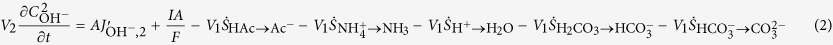


This equation shows that the hydroxide concentration in the catholyte is affected by the transport of hydroxide into/out of the catholyte (the first term on the right side), the production of hydroxide by Faradaic reactions (the second term on right), and various chemical reactions consuming or producing hydroxide (the remaining terms on right). The last two terms are hereafter lumped as 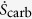. Figure 3c,d show the pH variation and the relative magnitude of each reaction term on the right-hand side of Eq. (2). We observe that, in the catholyte, the pH was predominately controlled by the Faradaic production of hydroxide and the consumption of hydroxide by their reaction with the NH_4_^+^ ions. Since there are few NH_4_^+^ ions inside the catholyte at the very beginning, the pH value rises rapidly during the earliest stage of operation. Unlike the NH_4_^+^ ions, the carbonate ions tend to increase the pH value because their chemical reactions inside the catholyte consume proton. Overall, the effect of carbonate ions on the pH in catholyte is limited, and this is related to their low concentration inside the catholyte. The latter is due to the limited carbonate ions inside the anolyte and their slow diffusion through the CEM. Since the pH regulation inside the catholyte depends strongly on the availability of NH_4_^+^ ions, and the latter in turn depends on the transport of NH_4_^+^ ions and NH_3_ across the CEM, it is clear that the pH regulation is intimately coupled with the transport of these species.

### Ammonium and ammonia transport

**Figure 4:**
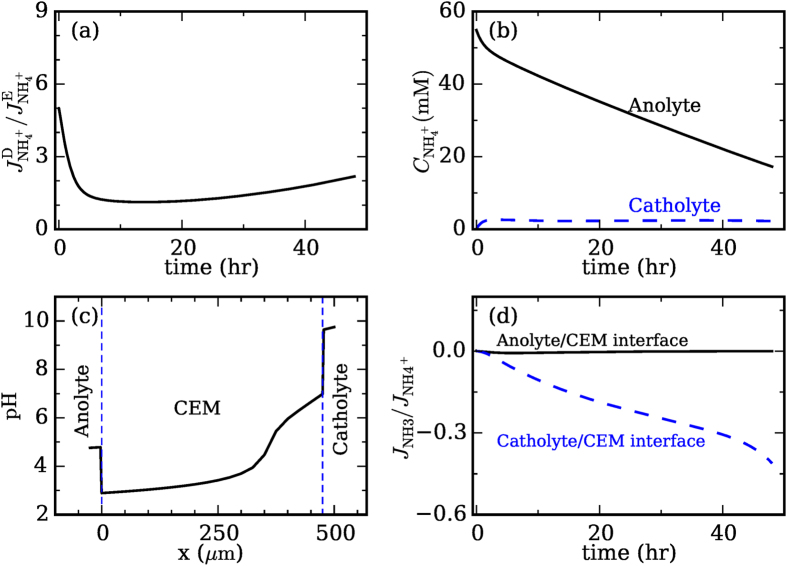
Mechanisms of NH_4_^+^/NH_3_ transport in MECs. (**a**) Relative strength of NH_4_^+^ ion transport due to diffusion and migration. (**b**) Evolution of the NH_4_^+^ ion concentration in the anolyte and catholyte. (**c**) Distribution of pH across the CEM and at the CEM-anolyte/catholyte interfaces. (**d**) The diffusion flux of NH_3_ toward the anolyte at the CEM-catholyte/anolyte interfaces. These diffusion fluxes are normalized by the total NH_4_^+^ ion flux to highlight their impact on the ammonia recovery in the catholyte. [ALL parameters in this study are the same as in Fig. 3.]

Such transport is analyzed from the perspective of how these species are removed from the anolyte. There are three ways to remove NH_3_/NH_4_^+^ ion from the anolyte: diffusion and migration of NH_4_^+^ ion through the CEM and the diffusion of NH_3_ through CEM. Figure 4a shows that, under the moderate electrical current considered here (I = 1 A m^−2^), the diffusion of NH_4_^+^ is actually stronger than its electrical migration. This somewhat surprising result is caused largely by the different pH value inside the anolyte and catholyte. As shown in Fig. 2, the anolyte (catholyte) is strongly acidic (basic) during operation. Hence, nitrogen element exists mostly as NH_4_^+^ in the anolyte and as NH_3_ in the catholyte (Fig. S1). Consequently, the NH_4_^+^ concentration in the anolyte is always much higher than that in the catholyte (Fig. 4b), which leads to a strong diffusion flux of the NH_4_^+^ ions. Since the acidic (basic) pH in the anolyte (catholyte) is ultimately induced by the electrical current through the system, we see that the imposed current serves two roles: it helps drive the migration of NH_4_^+^ ions from the anolyte toward catholyte, and it helps set up the pH environment that favors the diffusion of NH_4_^+^ ion from anolyte chamber toward the catholyte chamber. The second role has not been widely recognized. However, the fact that the diffusion of NH_4_^+^ ions dominates over their migration suggests that this second role is at least as important as the first role. We note that, while diffusion dominates the transport of NH_4_^+^ under low/moderate current densities (e.g., the I = 1 A m^−2^ considered here), electrical migration can become the dominant transport mechanism at high current densities (e.g., at I > 5 A m^−2^)^19^.

It is worth pointing out that, in absence of a net current, because of the low cation concentration in our catholyte (hence limited cation exchange between the CEM and catholyte), the removal of NH_4_^+^ from anolyte through transport across the CEM is minor. Indeed, both simulations and experiments suggest that ~2% of NH_4_^+^ could be removed from the anolyte in one batch cycle (48 hours), which is consistent with some prior studies^15^.

The dominance of NH_3_ over NH_4_^+^ ions inside the catholyte and the opposite trend in the anolyte implies that there can exist strong diffusion of NH_3_ from the catholyte chamber toward the anolyte chamber. Such diffusion can potentially lower the recovery efficiency of NH_3_ from the catholyte^20^. However, Fig. 4d shows that, although there is a relatively large amount of NH_3_ diffuse toward the anolyte chamber at the CEM-catholyte interface, very little NH_3_ crosses CEM-anolyte interface into the anolyte. This interesting phenomenon is caused by the pH environment within the CEM. As shown in Fig. 4d, within the CEM, the pH is strongly acidic at the anolyte side, which favors conversion of NH_3_ to NH_4_^+^ ions by 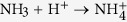 reaction. Hence, the anode side of the membrane served as the “reactor”: the NH_3_ diffused toward the anolyte chamber was turned into NH_4_^+^ within the membrane and subsequently diffused back into the cathode chamber.

More generally, the relevance of NH_3_ diffusion from catholyte to anolyte to its recovery can be estimated. The diffusion of NH_3_ is 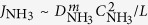 (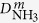: diffusion coefficient of NH_3_ in the membrane; 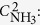 NH_3_ concentration in the catholyte; 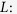
membrane thickness). The evaporating flux of NH_3_ in the catholyte is 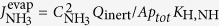
20,31. Hence, we define a dimensionless number


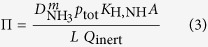


when 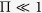, the effect of diffusion on ammonia recovery is negligible. For the system studied here, we have 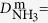

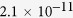
 m^2^/s, 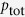 = 1 bar, 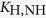 = 56250 mM/bar, L = 475 μm^32^, and aeration rate 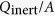 = 0.066 mol/(m^2^s). Hence, 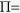
0.0376, i.e., the diffusion is weak enough to be ignored, which is consistent with the result in Fig. 4c. Since 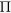 depends only on the design and operational parameters of the MEC, it can be used conveniently during the design of MECs.

### Ion Competition

**Figure 5:**
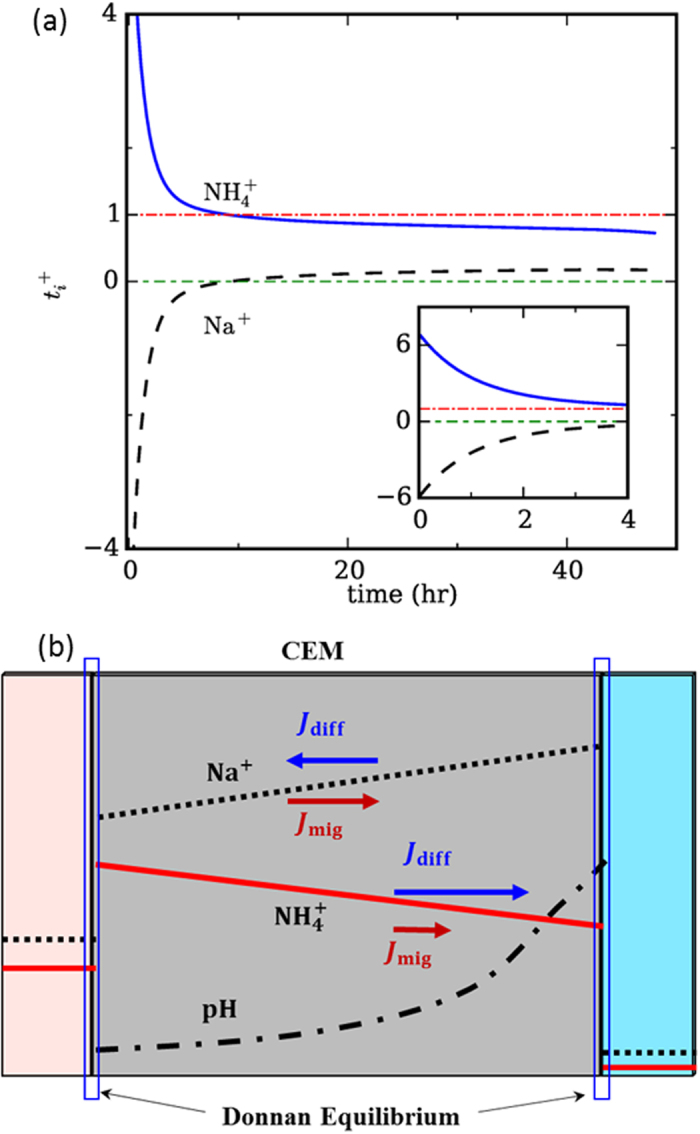
Ionic competition between Na^+^ and NH_4_^+^ . (**a**) Transport number of Na^+^ and NH_4_^+^ ions at the CEM-anolyte interface. (**b**) The concentration profiles and transport of Na^+^ and NH_4_^+^ ions within the cation exchange membrane. [ALL parameters in this study are the same as in Fig. 3.]

For a fixed current, various cations (e.g., Na^+^) in the anolyte can compete with the NH_4_^+^ ions for transport across the CEM, thus potentially compromising the removal of NH_4_^+^ ions from the anolyte. To quantitatively examine this competition, we computed the transport number of NH_4_^+^ and Na^+^ ions at the CEM-anolyte interface, i.e., 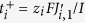 (Fig. 5a). At short time (*t* < 9 hr), the transport number of the Na^+^ ions is negative while that of the NH_4_^+^ ions is larger than 1.0, i.e., Na^+^ ions transport from the catholyte into the anolyte, and instead of competing with the NH_4_^+^ ions, they *facilitate* the removal of NH_4_^+^ ions from the anolyte. This phenomenon weakens with increasing time. At *t* > ~9 hr, Na^+^ ions do compete with NH_4_^+^ ions for transport across CEM, but the effect is still weak since the transport number of NH_4_^+^ ions is ~0.8 even at *t* = 40 hr.

The above surprising results are caused largely by the Donnan effects. Driven by the Donnan equilibrium, the concentrations of cations at membrane-anolyte/catholyte interfaces are elevated greatly above their concentrations in the adjacent anolyte/catholyte to balance the negative fixed charge in the CEM. For Na^+^ ions, their concentration at the CEM-catholyte interface is very high because there are few other cations in the catholyte competing with them to balance the fixed negative charge in the CEM (Fig. 5b). Meanwhile, the Na^+^ ions concentration at the CEM-anolyte interface is only moderately high because there are many NH_4_^+^ ions in the anolyte to cooperate with Na^+^ ions to balance CEM’s fixed charge. Consequently, the concentration of Na^+^ ions within the CEM decreases from the catholyte side toward the anolyte, despite that there are more Na^+^ ions in the anolyte than in catholyte. At short time, such a negative concentration gradient drives a strong diffusion flux, leading to the increase of the Na^+^ ion concentration in the anolyte. Because of current conservation, more NH_4_^+^ ions must be removed from the anolyte than that corresponding to the current passing out of the anolyte, thus leading to a facilitated NH_4_^+^ ion transport, i.e., 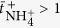. At longer time, the Na^+^ concentration at the CEM-anolyte interface increases (due to the decrease of NH_4_^+^ concentration in the anolyte) and the opposite happens at the CEM-catholyte interface. Hence, the diffusion of Na^+^ ions is weakened. Eventually, the diffusion of Na^+^ ions becomes weaker than their migration toward catholyte (Fig. 5b). However, since the migration is always partly canceled by the diffusion, Na^+^ ions always compete weakly with NH_4_^+^ ions for transport across the CEM. As a result, the current through the system is mostly carried by the NH_4_^+^ ions, which is consistent with our experimental data.

The above trends in ion competition are weakly modified when the initial concentration of Na^+^ ions in the anolyte changes. For higher initial concentration of Na^+^ ions, the diffusion of Na^+^ ions from catholyte to anolyte and its positive effect on NH_4_^+^ ions removal from anolyte during the early stage of operation decreases moderately, and the competition of Na^+^ ions for transport across CEM sets in earlier; for lower initial concentration of Na^+^ ions, the opposite occurs (see Figs S2 and S3).

## Discussion

Using integrated experimental and simulation studies, we examined the NH_4_^+^/NH_3_ transport in MECs and their coupling with the current generation, the acid-base reactions, and the transport of inert cations in the system. During MEC operation, a cascade of chemical groups regulates the pH in system to lead to an acidic (basic) environment in the anolyte (catholyte). The NH_4_^+^/NH_3_ couple is found to play a dual role in the operation: it serves as an effective proton shuttle for the charge transport across the CEM and also as buffer agent in the anolyte and catholyte. Inert cations, even in abundance, compete rather moderately with NH_4_^+^ ion for transport across the CEM. The strength of the diffusion of NH_3_ from catholyte to anolyte is governed by the MEC’s operating conditions and can be estimated using a dimensionless number.

The findings of this study will potentially impact the ammonia recovery using BES in several aspects. First, a clear understanding of ion transport and interaction will help interpret NH_4_^+^ ion recovery efficiency that may be affected by the composition of wastewater. Second, delineating the role of different chemical groups in pH regulation will help guide the BES operation for optimal pH. Third, the dimensionless number of diffusion of NH_3_ will help select suitable membranes. In this work, ammonium removal and recovery was examined under a high-current condition in BES, because high current generation was expected to benefit the transport of ammonium ions. Reducing current generation via lowering applied voltage and/or increasing external resistance would decrease ammonium removal/recovery.

There are still challenges to address in the future to improve the agreement between the model predictions and the experiment data. For example, there is a slight volume loss of the catholyte due to the aeration, and this effect was not considered in our model but it is likely to affect the concentration prediction in the catholyte to some extent. The bacteria activity will need to be considered in the future work. The bacteria activity in the anode could affect the balance of most of the species within the system, especially NH_4_^+^ and Na^+^, both of which are important to ion competition.

Future studies will focus on systematic investigation of the role of current in ion transport and ammonia recovery, the interaction between electric potential and ammonium recovery, improving the numerical model by integrating current generation models into the existing model, and extension of the unsteady model to other BES systems.

## Materials and Methods

### MEC setup

A bench-scale cubic shape MEC was used in this experiment. Both the anode and the cathode chambers had the same dimension of 9 cm × 4.7 cm × 0.8 cm. The liquid volume of the anode chamber was 200 mL, while that of the cathode chamber was 180 mL. A CEM (CMI-7000, Membrane International Inc., Glen Rock, NJ, USA) with a sectional area of 42.3 cm^2^ was used to separate anode and cathode chamber. The anode electrode was a carbon brush (Gordon Brush Mfg. Co., Inc., CA, USA) and the cathode electrode was a piece of 32-cm^2^ carbon cloth (Zoltek Companies, Inc., MO, USA) that was coated with 5 mg cm^−2^ of Pt/C (10% wt. Pt on Carbon Vulcan, Fuel Cell Earth LLC, USA). The anode was inoculated with the anaerobic sludge from the Peppers Ferry Regional Wastewater Treatment Plant (Radford, VA, USA). To mimic the digestion effluent of livestock waste^33^, the anode influent solution was prepared containing (per liter of deionized water): sodium acetate, 1.5 g; NH_4_Cl, 3.0 g; NaHCO_3_, 2.0 g; NaCl, 0.15 g; MgSO_4_, 0.005 g; CaCl_2_, 0.006 g; and trace elements solution^34^, 1 mL. The cathode chamber was initially filled with 180 mL of deionized water. An external voltage of 0.8 V was applied to the circuit by a power supply (CSI3644A, Circuit Specialists, Inc., Mesa, AZ, USA) according to a previous study^35^. The MEC was operated in a batch mode at room temperature (~20 °C). The anolyte was partially replaced (75%) every 48 h while the catholyte was resupplied to initial volume (180 mL) when a new batch cycle started. Both the anolyte and catholyte were recirculated at a flow rate of 20 mL min^−1^. The aeration rate was 375 mL min^−1^. 2 mL sample were collected regularly from both chambers for measurement and 1 M H_2_SO_4_ was used to absorb the stripped NH_3_ gas from cathode.

### Measurement and Analysis

The voltage across a 1-Ω resistor (R) in the MEC circuit was recorded every 2 min by a digital multimeter (2700, Keithley Instruments Inc., Cleveland, OH, USA). The pH of liquid stream was measured by two benchtop pH meters installed in the anode and the cathode, respectively (Oakton Instruments, Vernon Hills, IL, USA and Accumet AB250, Fisher Scientific, Pittsburgh, PA, USA). The concentrations of chemical oxygen demand (COD) and ammonium nitrogen (NH_4_^+^-N) were measured using a DR/890 colorimeter (HACH Co., Ltd., USA) according to manufacturer’s instruction. Ionic concentrations (e.g., Na^+^, Cl^−^) were quantified by using ion chromatography (Dionex LC20 ion chromatograph, Sunnyvale, CA, U.S.A.) equipped with an ED40 eletrochemical detector. The acetate concentration was measured in the anolyte and the catholyte samples that were filtered through 0.22 um PVDF membrane filter and by high-performance liquid chromatorgraphy (HPLC) (Shimadzu, Columbia, MD), equipped with an Aminex HPX-87H column (Bio-Rad, Hercules, CA) and refractive index detector (RID, 10A, Shimadzu). The HPLC column was kept at 65 °C, and 0.5 mM sulfuric acid solution was used as a mobile phase at flow rate of 0.6 mL min^−1^.

### Mathematical Model of BES-based Ammonia Recovery

We extend the previous steady state theory^20^ to model the ammonia recovery in BES operating in the batch mode. The model considers the transport of various species across the CEM and the acid-base reactions within the entire system. Without loss of generality, the following species are included: Na^+^, Cl^−^, HAc, Ac^−^, NH_4_^+^, NH_3_, H_2_CO_3_, HCO_3_^−^, CO_3_^2−^, H^+^ and OH^−^. The system is divided into three parts: the anode chamber, the cathode chamber, and the CEM (Fig. 1). The anode and the cathode chambers are both (but separately) treated in a lumped way, and the evolution of the average concentration of a species *i* inside each chamber follows


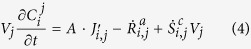


where 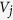 is the volume of chamber *j* (*j* = 1: anode chamber; *j* = 2 cathode chamber), 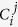 is the concentration of species *i* in chamber *j*, 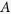 is the area of the CEM, and 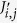 is the flux of species *i* into chamber *j*. 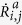 is the removal of species *i* (e.g., CO_2_, NH_3_ etc., acetate removal due to the aeration is neglected, since it’s concentration in the catholyte is always low, as shown in the study) from chamber *j* due to aeration, which is determined by assuming fast equilibrium between species dissolved in the catholyte and existing in the aeration gas. Previous study had shown that this assumption works well^20^,^31^. For example, the removal of 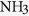 from the cathode chamber, 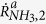, is given by 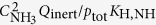, where 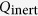 and 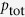 are the volumetric flow rate and pressure of the inert aeration gas, respectively. 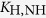 is the Henry’s constant for 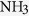 in water. 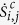 is the generation/removal rate (per volume) of the species *i* due to chemical/biological reactions, respectively. Briefly, we consider the consumption of Ac^−^ by the microbes, the acid-base equilibrium among different species (e.g., 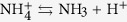, 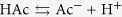, …). A list of all reactions considered can be found in Table S1 in the Supplementary Information. According to the previous work^20^, we assume that the acid-base reactions are fast so that the chemical equilibrium between the species involved in these reactions is always maintained.

The CEM is resolved spatially in its thickness direction (*x* = 0 and *L* correspond to the CEM-anolyte and CEM-catholyte interfaces, respectively). Each species *i* at *x* = 0 and *L* is always in equilibrium with that in the anode and cathode chambers, respectively (see below for more details). The distribution of each species *i* across the CEM, 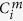, is governed by


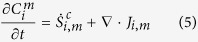


where 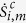 is the generation/consumption of species *i* by acid-base equilibriums and is determined in the same way as in the electrode chambers. 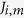 is the flux of species *i* in the membrane given by the Nernst-Planck equation^20^,^36^,^37^:


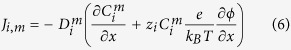


where 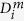 and 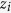 are the diffusion coefficient in the membrane and valence of species *i*, respectively; 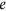 and 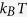 are the electron charge and thermal energy; 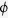 is the electrical potential. Because the concentration of various species inside the anode/cathode chambers evolves slowly at a time scale of tens of hours but the relaxation of species concentration within the CEM is fast due to CEM’s small thickness, the time dependence term in Eq. (5) is unimportant and thus dropped hereafter.

The concentration distribution of any species *i* across the anolyte/catholyte-CEM interfaces is treated as follows. For electrically neutral species, their concentrations are continuous across these interfaces. For a charged species *i*, we adopt the Donnan equilibrium condition^20^,^36^,^38^, i.e.,


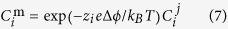


where 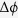 is the electrical potential drop across the CEM-solution interface (usually termed the Donnan potential). Note that the Donnan potential attract cations into (repel anions from) the CEM so that the negative fixed charge inside the membrane is balanced by the free ions.

Finally, the electro-neutrality condition and the charge conservation law are enforced:


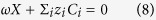



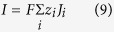


where 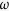 is the sign of the fixed space charge in any domain (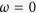 in the anode/cathode chamber and zero charge membranes; 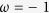 and +1 in cation and anion exchange membranes, respectively). 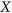 is the fixed charge density of each domain. 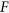 and 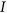 are the Faraday constant and the current density through the CEM.

Equations (4, 5, 6, 7, 8, 9), along with the equations for various chemical reactions, form a complete model of the ammonia recovery operation using BES. These nonlinear, time-dependent equations were solved using MATLAB. In our simulations, all design and operation parameters of the MEC, e.g., the volume of electrode chamber, aeration rate, and current density (Fig. 2a), are taken from that in the experiments unless otherwise mentioned. For the CEM (CMI-7000) used in this system, 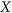 was determined to be 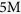 using the method established in prior studies^20^,^32^,^37^,^39^. The diffusion coefficient of various species in the CEM is usually smaller than that in bulk solutions^20^,^40^. The diffusion coefficients of all species are either taken from prior experimental data or by fitting the current experimental results. In the latter case, care was taken to ensure that the fitted data are within the range expected for ion diffusion in typical CEMs^9^,^20^,^21^,^41^. See Tables S2–S4 for a summary of all parameters used in these simulations.

## Additional Information

**How to cite this article**: Liu, Y. *et al*. Understanding Ammonium Transport in Bioelectrochemical Systems towards its Recovery. *Sci. Rep.* **6**, 22547; doi: 10.1038/srep22547 (2016).

## Supplementary Material

Supplementary Information
