# Supplementary material for: Understanding Ammonium Transport in Bioelectrochemical Systems towards its Recovery
Source: Sci Rep. 2016 Mar 3;6:22547. doi: 10.1038/srep22547 (PMC4776096; doi:10.1038/srep22547)
Supplement: Supplementary Information [file srep22547-s1.pdf]

**Supplementary Information**

# Understanding Ammonium Transport in Bioelectrochemical Systems toward its Recovery

Ying Liu, [1,†] Mohan Qin, [2,†] Shuai Luo, [2] Zhen He, [2,*] Rui Qiao[1,*]

[1] *Department of Mechanical Engineering, Virginia Polytechnic Institute and State University, Blacksburg, VA 24061, USA*

[2] *Department of Civil and Environmental Engineering, Virginia Polytechnic Institute and State University, Blacksburg, VA 24061, USA*

## 1. Modeling of electrochemical and chemical reactions in the MEC

Because of bacteria activity, acetate ($Ac^-$) is oxidized on the anode to release electron and proton:

$$\mathrm{Ac^- + 4H_2O \rightarrow 2HCO_3^- + 9H^+ + 8e^-} \quad \text{(S1)}$$

Through the external circuit, the released electrons are transferred to the cathode and react with the oxygen from the aeration:

$$\mathrm{4e^- + O_2 + 2H_2O \rightarrow 4OH^-} \quad \text{(S2)}$$

Since both equations are directly related to the electron transfer, the consumption/production of related species within the system can be modeled as a function of current, and they are referred to as Faradaic consumption/production in the main text.

The concentration of a species in the anolyte/catholyte/membrane can change due to its transport, Faradaic reactions (if applicable) and acid-base type chemical reactions. The effects of the acid-

base type reactions are modeled as a sink/source term. Here, we illustrate the treatment of these acid-base type reactions using the evolution of $NH_4^+/NH_3$ concentration inside the anolyte as an example. For this couple, their reaction $\mathrm{NH_3 + H^+ \leftrightarrows NH_4^+}$ and their transport flux collectively change their concentrations. In the anolyte, the evolution of their concentrations is governed by

$$V_1 \frac{\partial C^1_{\mathrm{NH_4^+}}}{\partial t} = A \cdot J'_{\mathrm{NH_4^+},1} + V_1 \dot{S}^c_{\mathrm{NH_3 \to NH_4^+}} \tag{S3}$$

$$V_1 \frac{\partial C^1_{\mathrm{NH_3}}}{\partial t} = A \cdot J'_{\mathrm{NH_3},1} - V_1 J^c_{\mathrm{NH_3},1} \tag{S4}$$

where $J'_{\mathrm{NH_4^+},1}$ and $J'_{\mathrm{NH_3},1}$ are transport of $NH_4^+$ and $NH_3$ into the anolyte through the CEM, respectively. $V_1 \dot{S}_{\mathrm{NH_3 \to NH_4^+}}$ represents the amount of $NH_3$ converted to $NH_4^+$ due to the reaction $\mathrm{NH_3 + H^+ \leftrightarrows NH_4^+}$ . Combing Eq.(S3) and (S4) gives a new equation lumping the $NH_4^+$ and $NH_3$ together.

$$V_1 \frac{\partial C^1_{\mathrm{NH_4^+}}}{\partial t} + V_1 \frac{\partial C^1_{\mathrm{NH_3}}}{\partial t} = A \cdot (J'_{\mathrm{NH_4^+},1} + J'_{\mathrm{NH_3},1}) \tag{S5}$$

Assuming fast equilibrium for the acid-base reaction, the partition between the $NH_4^+$ and $NH_3$ , follows:[1-4]

$$K_{\mathrm{a,NH}} = \frac{C^1_{\mathrm{NH3}} \cdot C^1_{\mathrm{H^+}}}{C^1_{\mathrm{NH_4^+}}} \tag{S6}$$

where $K_{\mathrm{a,NH}}$ is the chemical equilibrium constant for the reaction $\mathrm{NH_3 + H^+ \leftrightarrows NH_4^+}$. Equation S6 states that the ratio of the $NH_4^+$ and $NH_3$ concentrations are directly related to the pH locally. As shown in Fig. S1, for pH<7 $NH_4^+$ counts more than 99.4% of the total ammonium/ammonia; for pH>9.5, $NH_3$ counts more than 64%. Similar mathematical treatment is applied to all other acid-base type reactions. A full list of the acid-base reactions and their equilibrium constant is provided in Table S2.

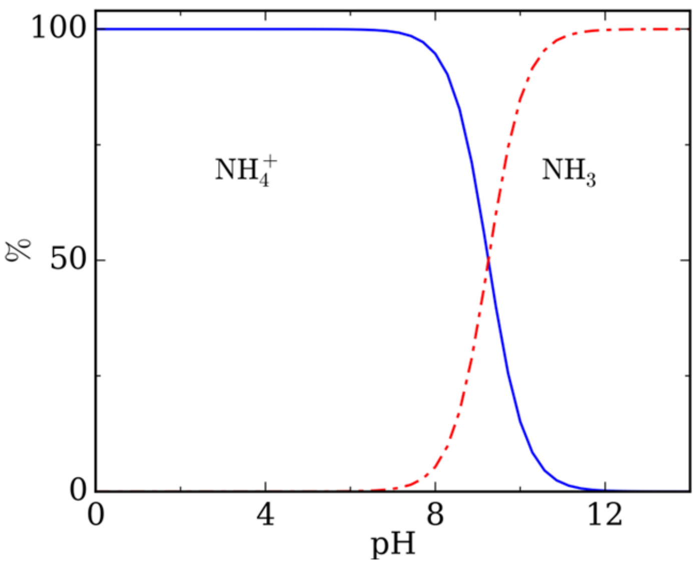


**Fig. S1**: Partition between $NH_4^+$ and $NH_3$ as a function of pH assuming fast $NH_3 + H^+ \leftrightarrows NH_4^+$ reaction.

## 2. Governing equation for $H^+$ ion concentration in the anolyte

Equation 7 in the main text shows how the $H^+$ ion concentration in the anolyte evolves a function of time. Here we elaborate on the meaning of each term in the equation and how it is evaluated computationally. We note that there are several possible ways to change the $H^+$ ion concentration in anolyte: the flux of proton into/out of the anode chamber, the Faradaic reaction due to the bacteria oxidation, and the consumption/production due to reaction with other species, which include all the acid-base reactions shown in Table S1. Using conservation law, Equ. 7 is obtained as (reproduced here for convenience)

$$V_1 \frac{\partial C^1_{\mathrm{H^+}}}{\partial t} = A \cdot J'_{H^+,1} + \frac{9}{8}\frac{IA}{F} - V_1 \dot{S}_{\mathrm{NH_3 \to NH_4^+}} - V_1 \dot{S}_{\mathrm{Ac^- \to HAc}} - V_1 \dot{S}_{\mathrm{OH^- \to H_2O}} - V_1 \dot{S}_{\mathrm{HCO_3^- \to H_2CO_3}} + V_1 \dot{S}_{\mathrm{HCO_3^- \to CO_3^{2-}}} \quad \text{(S7)}$$

As explained in Equs. S3 and S4, $V_1 \dot{S}_{\mathrm{NH_3 \to NH_4^+}}$ stands for the conversion of $NH_3$ to $NH_4^+$ by the reaction $NH_3 + H^+ \to NH_4^+$, which equals to the consumption of proton. This term can be calculated by manipulating Equ. S4, giving $V_1 \dot{S}_{\mathrm{NH_3 \to NH_4^+}} = -(V_1 \frac{\partial C^1_{\mathrm{NH_3}}}{\partial t} - A \cdot J'_{\mathrm{NH_3},1})$; Using the

$J'_{NH_3,1}$ calculated in simulations, $V_1\dot{S}_{NH_3 \to NH_4^+}$ can be evaluated. Similar process could be applied to all other source/sink terms in Equ. 7 in the main text and the results are as follows:

$V_1\dot{S}_{Ac^- \to HAc} = V_1\frac{\partial C^1_{HAc}}{\partial t} - A \cdot J'_{HAc,1}$, $V_1\dot{S}_{HCO_3^- \to H_2CO_3} = V_1\frac{\partial C^1_{H_2CO_3}}{\partial t} - A \cdot J'_{H_2CO_3,1}$, $V_1\dot{S}_{HCO_3^- \to CO_3^{2-}} = V_1\frac{\partial C^1_{CO_3^{2-}}}{\partial t} - A \cdot J'_{CO_3^{2-},1}$, $V_1\dot{S}_{OH^- \to H_2O} = -(V_1\frac{\partial C^1_{OH^-}}{\partial t} - A \cdot J'_{OH^-,1})$. All variables are named under the same convention as in the main text, i.e., $C_i^1$ stands for the concentration of species *i* in the anolyte and $J'_{i,j}$ is the flux of species *i* into chamber *j* (*j*=1: anode chamber; *j*=2 cathode chamber).

## 3. Model parameters

**Table S1. Equilibrium constants for acid-base reactions and aqueous-solution equilibrium**

| | Constant | Reaction | Equilibrium equation | Value* | Ref. |
|---|---|---|---|---|---|
| Acid-base reaction | $K_{a,Ac}$ | $HAc \leftrightarrow Ac^- + H^+$ | $K_{a,Ac} = \frac{C_{Ac^-} \cdot C_{H^+}}{C_{HAc}}$ | 0.0174 | 2 |
| | $K_{a,CA1}$ | $H_2CO_3 \leftrightarrow H^+ + HCO_3^-$ | $K_{a,CA1} = \frac{C_{H^+} \cdot C_{HCO_3^-}}{C_{H_2CO_3}}$ | $4.4668\times10^{-4}$ | 2 |
| | $K_{a,CA2}$ | $HCO_3^- \leftrightarrow H^+ + CO_3^{2-}$ | $K_{a,CA2} = \frac{C_{H^+} \cdot C_{CO_3^{2-}}}{C_{HCO_3^-}}$ | $4.6774\times10^{-8}$ | 2 |
| | $K_{a,NH}$ | $NH_4^+ \leftrightarrow NH_3 + H^+$ | $K_{a,NH} = \frac{C_{H^+} \cdot C_{NH_3}}{C_{NH_4^+}}$ | $5.6234\times10^{-7}$ | 2 |
| | $K_w$ | $H_2O \leftrightarrow H^+ + OH^-$ | $K_w = C_{H^+} \cdot C_{OH^-}$ | $1\times10^{-8}$ | 2 |
| Henry's constant | $K_{H,CO_2}$ | | $K_{H,CO_2} = \frac{C_{CO_2,aq}}{p_{CO_2}}$ | 33.46 | 2,3 |
| | $K_{H,NH_3}$ | | $K_{H,NH_3} = \frac{C_{NH_3,aq}}{p_{NH_3}}$ | 56250 | 2 |

*The values correspond to concentration unit of mol/m$^3$ and pressure unit of atm;

**Table S2. System configuration**

| Parameters | Values | Notes |
|---|---|---|
| A ($m^2$) | $42.3\times10^{-4}$ | Membrane area |
| $V_1$ ($m^3$) | $0.2\times10^{-3}$ | Volume of anode chamber |
| $V_2$($m^3$) | $0.18\times10^{-3}$ | Volume of cathode chamber |
| L (m) | $4.75\times10^{-4}$ | Membrane thickness |
| X (M) | 5 | Membrane's fixed charge concentration (CEM-7000) |
| $J_{\mathrm{inert}}$ (mol $m^{-2}s^{-1}$) | $6.6\times10^{-2}$ | Aeration rate |
| $t_0$ (hr) | 48 | Operation time of each batch cycle |

**Table S3. Diffusion coefficients of all species**

| Species | in free solution ($\times10^{-9}$ $m^2s^{-1}$) | in CEM ($\times10^{-9}$ $m^2s^{-1}$) | Comment |
|---|---|---|---|
| $Na^+$ | 1.33 | 0.1064 | |
| $Cl^-$ | 2.02 | 0.1056 | |
| HAc | 1.21 | 0.0067 | Ref. 5 |
| Ac- | 1.10 | 0.0110 | |
| $NH_4^+$ | 1.94 | 0.0795 | |
| $NH_3$ | 2.10 | 0.0210 | |
| $H_2CO_3$ | 1.92 | 0.1536 | |
| $HCO_3^-$ | 1.18 | 0.0354 | |
| $CO_3^{2-}$ | 0.98 | 0.0294 | |
| $H^+$ | 9.13 | 0.3296 | |
| $OH^-$ | 5.16 | 0.1481 | |

**Table S4. Initial conditions in the anolyte and catholyte in numerical simulations**

| Species | Concentration in anolyte (mM) | Concentration in catholyte (mM)* | Comment |
|---|---|---|---|
| $Na^+$ | 45.199 | $1.000\times10^{-5}$ | 10mM in catholyte in the parametric study |
| $Cl^-$ | 58.834 | $1.000\times10^{-4}$ | |
| HAc | $2.597\times10^{-2}$ | $9.517\times10^{-7}$ | |
| Ac- | 18.259 | $9.962\times10^{-5}$ | |
| $NH_4^+$ | 54.837 | $1.002\times10^{-4}$ | |
| $NH_3$ | 1.248 | $3.396\times10^{-7}$ | |
| $H_2CO_3$ | 1.264 | $6.077\times10^{-6}$ | 3.203mM in catholyte in parametric study |
| $HCO_3^-$ | 22.856 | $1.635\times10^{-5}$ | 9.993mM in catholyte in parametric study |
| $CO_3^{2-}$ | $4.326\times10^{-2}$ | $4.720\times10^{-9}$ | $3.264\times10^{-3}$mM in catholyte in parametric study |
| $H^+$ | $2.471\times10^{-5}$ | $1.660\times10^{-4}$ | |
| $OH^-$ | $4.047\times10^{-4}$ | $6.024\times10^{-5}$ | |

*The initial species concentration in catholyte is mostly practically 0 in the validation simulation, the same as in the experiment. In the parametric study, the initial species concentration is the same unless otherwise denoted.

## 4. Ionic competition

As explained in the main text, due to the opposite direction of their diffusion and migration through the CEM, $Na^+$ carries little charge across the CEM and the ion competition effect between $Na^+$ and $NH_4^+$ is moderate. Here we assess the ion competition effect when the $Na^+$ ion concentration inside the anolyte is even higher than that of the $NH_4^+$ ions. As shown in Fig. S2, for higher initial concentration of $Na^+$ ions, the backward transport of $Na^+$ ions from the catholyte into the anolyte and associated positive effect on $NH_4^+$ ions removal from the anolyte

during the early stage of operation decreases moderately. The competition of $Na^+$ ions for transport across CEM sets in earlier than that under lower $Na^+$ ion concentration in the anolyte. For lower initial concentration of $Na^+$ ions, the opposite occurs.

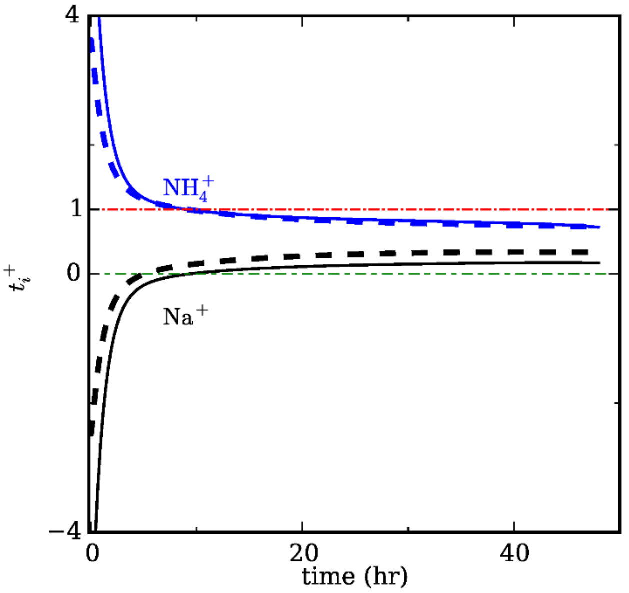


**Fig. S2**: Transport number of $Na^+$ and $NH_4^+$ ions at the CEM-anolyte interface under different initial $Na^+$ ion concentration in the anolyte. The solid (dashed) lines are for case in which the initial concentration of $Na^+$ ions in the anolyte is equal to (three times of) that of the $NH_4^+$ ions.

To further assess the ion competition effect, we examined the $NH_4^+$ concentration in anolyte during MEC operation with different initial $Na^+$ concentration in the anolyte while keeping all other operating conditions the same. Figure S3 shows that, as the initial $Na^+$ concentration in the anolyte increases (decreases), less (more) $NH_4^+$ ions are transported out of the anolyte due to stronger ion competition. Most of difference occurs during the early stage of operation, and the difference in the later stage is small. Overall, the transport of $NH_4^+$ ions out of the anolyte is affected only moderately by the initial $Na^+$ ion concentration in the anolyte, suggesting that the ion competition effect is moderate in the system studied here.

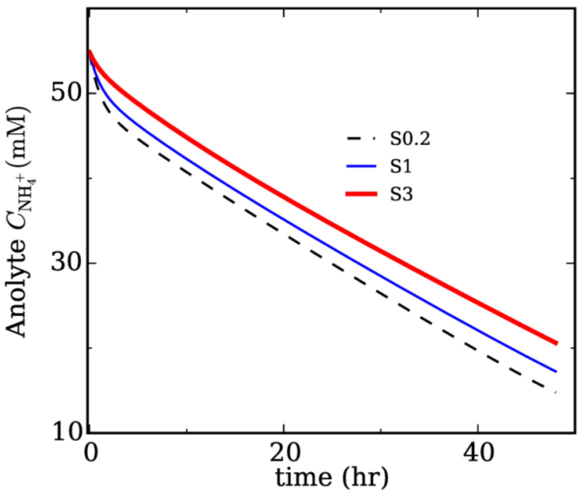


**Fig. S3**: Evolution of the $NH_4^+$ ion concentration in anolyte during operations with different initial $Na^+$ ion concentrations in the anolyte. S0.2, S1, and S3 corresponds to the case in which the initial $Na^+$ ion concentration in the anolyte is 0.2, 1.0 and 3.0 (for demonstration purpose) times of the initial $NH_4^+$ ion concentration, respectively. The operating parameters (e.g., current density and aeration rate) are otherwise identical in all cases.

## Symbols and Abbreviations

| | |
|---|---|
| $C_i^j$ | Concentration of species *i* in chamber *j* |
| $D_i^m$ | The diffusion coefficient of species *i* in the membrane |
| $e$ | Elementary charge |
| $F$ | Faraday constant, 96485 C/mol |
| $I$ | The current density normalized to the membrane area |
| $J'_{i,j}$ | Flux of species *i* into chamber *j* |
| $J_{i,m}$ | Flux of species *i* within the membrane |
| $J_i^D$ | Diffusion flux of species *i* |
| $J_i^E$ | Electrical migration flux of species *i* |
| $p_{\mathrm{tot}}$ | The pressure of the inert aeration gas |
| $Q_{\mathrm{inert}}$ | The volumetric flow rate of the inert aeration gas |
| $Q_{NH_3}$ | The recovery rate of $NH_3$ from the catholyte due to the aeration |
| $\dot{R}_{i,j}^a$ | Removal of species *i* (e.g., $CO_2$, $NH_3$ etc., acetate removal due to the aeration is neglected, since it's concentration in the catholyte is always low, as shown in the study) from chamber *j* due to aeration |

| | |
|---|---|
| $\dot{S}_{i,j}^{c}$ | The generation/removal rate (per volume) of the species $i$ due to chemical/biological reactions in chamber $j$ |
| $\dot{S}_{i,m}^{c}$ | Generation/consumption of species $i$ by acid-base equilibriums |
| $t$ | time |
| $\bar{t}_{\mathrm{Na}^+}^{+}$ | Cumulative transport number of species $i$ |
| $V_j$ | Volume of the anolyte and catholyte chamber $j$ ($j$=1: anode chamber; $j$=2: cathode chamber) |
| $x$ | Position across the membrane ($x$=0 and $L$ correspond to the CEM-anolyte and CEM-catholyte interfaces, respectively) |
| $X$ | Fixed charge density of each domain: $X = 5$ M for the CEM (CMI-7000) |
| $z_i$ | The valence of species $i$ in the membrane |
| $k_B T$ | Thermal energy |
| $\phi$ | Electrical potential |
| $\omega$ | Sign of the fixed space charge in domain: $\omega = 0$ in the anode/cathode chamber and zero charge membranes; $\omega = -1$ and +1 in cation and anion exchange membranes, respectively |

Subscript and superscript

| | |
|---|---|
| $i$ | chemical species |
| $j$ | chamber $j$, $j$=1: anode chamber; $j$=2: cathode chamber |
| $m$ | membrane |

## References


1 Kuntke, P. *et al.* Ammonium recovery and energy production from urine by a microbial fuel cell. *Water Res.* **46**, 2627-2636 (2012).

2 Dykstra, J. E., Biesheuvel, P. M., Bruning, H. & Ter Heijne, A. Theory of ion transport with fast acid-base equilibrations in bioelectrochemical systems. *Physical Review E* **90**, 013302 (2014).

3 Persat, A., Chambers, R. D. & Santiago, J. G. Basic principles of electrolyte chemistry for microfluidic electrokinetics. Part I: acid–base equilibria and pH buffers. *Lab on a Chip* **9**, 2437-2453 (2009).

4 Powers, S. E., Collins, A. G., Edzwald, J. K. & Dietrich, J. M. Modeling an aerated bubble ammonia stripping process. *J. - Water Pollut. Control Fed.* **59**, 92-100 (1987).

5 Kim, J. R., Cheng, S., Oh, S. E. & Logan, B. E. Power generation using different cation, anion, and ultrafiltration membranes in microbial fuel cells. *Environ. Sci. Technol.* **41**, 1004-1009 (2007).
